# Supplementary material for: Self-determination theory in acute child and adolescent mental health inpatient care. A qualitative exploratory study
Source: PLoS One. 2020 Oct 16;15(10):e0239815. doi: 10.1371/journal.pone.0239815 (PMC7567378; doi:10.1371/journal.pone.0239815)
Supplement: Attachment — Submitted filename: Hp Response to reviewers final.docx [file pone.0239815.s001.docx]

**Comments to the Author**

1. Is the manuscript technically sound, and do the data support the conclusions?

The manuscript must describe a technically sound piece of scientific research with data that supports the conclusions. Experiments must have been conducted rigorously, with appropriate controls, replication, and sample sizes. The conclusions must be drawn appropriately based on the data presented.

Reviewer #1: Yes

Reviewer #2: Partly

2. Has the statistical analysis been performed appropriately and rigorously?

Reviewer #1: N/A

Reviewer #2: N/A

3. Have the authors made all data underlying the findings in their manuscript fully available?

The [PLOS Data policy](http://scanmail.trustwave.com/?c=7264&d=lJjH3lp2NKshGw06EGQNC8fZ13yPEeuEu0IkIrTH2g&u=http%3a%2f%2fwww%2eplosone%2eorg%2fstatic%2fpolicies%2eaction%23sharing) requires authors to make all data underlying the findings described in their manuscript fully available without restriction, with rare exception (please refer to the Data Availability Statement in the manuscript PDF file). The data should be provided as part of the manuscript or its supporting information, or deposited to a public repository. For example, in addition to summary statistics, the data points behind means, medians and variance measures should be available. If there are restrictions on publicly sharing data—e.g. participant privacy or use of data from a third party—those must be specified.

Reviewer #1: No

Reviewer #2: Yes

4. Is the manuscript presented in an intelligible fashion and written in standard English?

PLOS ONE does not copyedit accepted manuscripts, so the language in submitted articles must be clear, correct, and unambiguous. Any typographical or grammatical errors should be corrected at revision, so please note any specific errors here.

Reviewer #1: Yes

Reviewer #2: Yes

5. Review Comments to the Author

Please use the space provided to explain your answers to the questions above. You may also include additional comments for the author, including concerns about dual publication, research ethics, or publication ethics. (Please upload your review as an attachment if it exceeds 20,000 characters)

Reviewer #1: Thank you for this lovely manuscript on an important topic.

Following are my minor revisions:

On line 35 and on line 557 please remove the formulation "may be more effective than talking or medication therapy". This conclusion can not be drawn by this study design and can not stand as it is.

*Thank you for clarifying this. We have removed this conclusion.*

Move from line 79 to 106 to Methods.

*While appreciating this recommendation we feel that having the description of the unit in the introduction contributes to the readability of the paper. The description of the unit and the process of change there is important in describing why we undertook the study.*

Add reference on line 129 after illness.

Add reference on line 131 after central.

*We have rewritten these two sentences to be more specifically evidence based and included appropriate references.*

*“(Ryan & Deci, 2000). Lack of motivation is a key part of the negative symptom complex associated with limited recovery from psychotic illness (Albert et al., 2011; Strauss, Harrow, Grossman, & Rosen, 2010). Developing motivation to work towards a life worth living is an essential aspect of therapy for suicidal adolescents (Miller, Rathus, & Linehan, 2007)*”

On line 183 add median in minutes.

*We do not have a record of the median.*

On line 445 change "re" to "in".

*Thanks, we have made this change*

Erase: line 534 to 553 irrelevant to the topic.

*Thanks for the recommendation, we have removed these lines.*

Erase line 564 to 565 This offers...

*Thanks again, we realised we have not made the relevance clear and have rewritten the section.*

*“Activities which meet needs for autonomy, relatedness and competence could be further explored in the community context as a possibility for a therapeutic intervention for the many young people who are difficult to engage in talking or medication therapy.”*

Edit reference Smith-Gowling on line 572

*Thanks, we have done that.*

Change on line 598 "can" to "may".

*Thanks, we have done that.*

Reviewer #2: This study sheds light on the sensitive and important issue of children and adolescents in MH psychiatric facilities. As such it brings up an ongoing problem that is under addressed and sought in research. It makes an important contribution. However, the manuscript needs to be enhanced in its quality and revised.
Here are some points according to the sections of the article:

Introduction:

1.More knowledge to highlight the problem at first re the traumatic impact of hospitalization of mental health patients is needed. There has definitely been quite some work around that with adult manta health care that can be relevant to emphasize the need to address this problem.
Fror example, there are studies that specifically showed traumatization from psychiatric hospitalization at least in adults - which may be worth noting. For example see:
D. Paksarian, R. Mojtabai, R. Kotov, B. Cullen, K.L. Nugent, E.J. Bromet (2014). Perceptions of hospitalization-related trauma and treatment participation among individuals with psychotic disorders, Psychiatr Serv. 65(2): 266–269. doi: 10.1176/appi.ps.201200556

*Thanks for this, we have added this information with appropriate references.*

*“Harmful effects of traumatic, humiliating and coercive experiences have been documented in adult mental health inpatient care (Cusack, Frueh, Hiers, Suffoletta-Maierle, & Bennett, 2003; Paksarian et al., 2014).”*

2.The authors might be interested to describe more about the role of providers and the link between non-specific factors and support for recovery in mental illness, which has been researched at least with the adult population. For example:
Moran, G.S., Mashiach-Eizenberg, M., Roe, D., Berman, Y., Shalev, A., Kaplan, Z., & Epstein, P.G. (2014). Investigating the anatomy of the helping relationship in the context of psychiatric rehabilitation: The relation between working alliance, providers’ recovery competencies and personal recovery. Psychiatry Research, 220(1-2), 592-597
For an example of a focus on enhancing person-centered approach and recovery oriented interventions see:
Hornik‐Lurie, T., Shalev, A ., Haknazar, L., Garber Epstein, P., Ziedenberg‐Rehav, L., Moran, G. S. (2018). Implementing recovery‐oriented interventions with staff in a psychiatric hospital: A mixed‐methods study. Journal of Psychiatric and Mental Health Nursing, 25(9-10), 569-581
Another earlier work has focused on eliminating restraint and seclusion in a psychiatric facility that might be of interest is:
Ashcraft, L., Anthony, W. (2008). Eliminating seclusion and restraint in recovery-oriented crisis services. Psychiatric Services 59(10):1198-202. doi: 10.1176/appi.ps.59.10.1198.

*We appreciate the whole field of non-specific factors in recovery and elimination and restraint are important issues in inpatient mental health care but we feel that discussion and review of these is outside the focus of this paper.*

3. Please specify what DHB (and other acrynoms appearing in the text) stands for.

*Thanks for that, we have done that.*

4. Deci & Ryan conducted their studies in a broad range of settings and populations, it would be interesting to discern if and what were those studies that referred only to mental health populations. If there aren’t such studies, then the next sentence needs to be modified:
The core research outcome in many of the studies is intrinsic motivation rather than recovery from mental illness but fulfilling these needs significantly influences a 128 person’s performance and well-being at work and school (Ryan & Deci, 2000).
As currently it misleads the reader regarding Ryan & Deci’s work as if it refers directly to recovery in mental health.

*Thank you for this, we have clarified that Ryan and Deci’s work was largely done outside of mental health.*

*“The core research outcome in many of the studies is intrinsic motivation rather than recovery from mental illness and there is limited research done in mental health settings. But they have found in other settings that fulfilling these needs significantly influences a person’s performance and well-being at work and school (Ryan & Deci, 2000).”*

Methods:
More detail is needed.
1.For example, It is not clear when the interviews were taken and where.

*Thanks for pointing this out. We have clarified they were done in hospital.*

[2.In](http://scanmail.trustwave.com/?c=7264&d=lJjH3lp2NKshGw06EGQNC8fZ13yPEeuEu0MiIL7C2w&u=http%3a%2f%2f2%2eIn) addition, if the interviews were conducted during the time of hospitalization, more information needs to be provided regrading ethical considerations of the person’s condition.

*Indeed the NZ Ministry of Health, Ethics and Disability Committee had a lot of concern about this issue, as did we. We developed a multi-step consent process. We have further clarified the steps and that each step was sequential.*

*“Young people were initially interviewed in hospital during their acute admission. Because of their vulnerability at this time we worked with the Ethics Committee to develop a multi-step process of recruitment and informed consent. Steps were sequential and each step would need to be completed before the next could be undertaken. The steps were: (i) as part of their routine clinical assessments of the young people admitted to the unit the consultant Child Psychiatrists would assess them for competence to consent to the research. (ii) Once the young person was considered competent the clinician would ask their parents (or guardians) if they would like to meet the research interviewer (MJ) to find out about the study. (iii) The research interviewer informed the parents or guardians, both orally and with written information, about the study and sought their written consent to approach their child. (iv) The research interviewer then approached the young person and informed them about the study with written (with an information sheet fitted to their chronological age and facility with reading) and oral information. He then sought their written consent to participate in the study. The researcher was not part of the clinical team. The young person and family were assured that their decision to consent or not would not be known by the treating team and would not affect their treatment.”*

3.Interview guide: please state the rationale for the questions in the interview guide, and also describe the interview process (where it took place, how long on average interviews were? Etc.)

*We have clarified the rationale for the questions in the interview guide. We have included the range of length of interviews but do not have the median or average available.*

4. If possible, please describe the number of pages of transcript produced from the 15 interviewees to give a sense of the amount of the materials analyzed.

*We have included this*

Data Analysis:
1.Please describe how incidences of disagreement between coders of the interviews were addressed in the analytic process.

*We have included this.*

*“Disagreements between coders throughout the whole analysis process were treated as an opportunity to access further depth in our understandings by clarifying the nature of the disagreements and what they were based on. This process contributed to the development of the coding in that it brought forward different aspects of the data.*

2. Given that you are relying on SDT as a conceptual guide in your analysis, perhaps you would like to address “theory driven approach to qualitative studies”, and how your work addressed relevant issues. (see for example : Macfarlane A, O'Reilly-de Brún M. (2012). Using a theory-driven conceptual framework in qualitative health research. 22(5):607-18. doi: 10.1177/1049732311431898.)

*Thank you for identifying this article. We have found it very helpful, have drawn from it and included it as a reference.*

*“The initial analysis by the whole study team, most of whom were only minimally familiar with Self-determination Theory, optimized the possibility that aspects of the data which did not resonate with Self-determination Theory could be brought forward, thus making it less likely that we were forcing the data into predetermined categories (MacFarlane & O'Reilly-de Brun, 2012).”*

Results
1.The opening statement needs to be improved to convey to the reader you are first presenting and overview of the findings, and then will provide elaborations with quotes for each theme.

*Thank you, we agree it is important the structure of the results is clear and have added a heading indicating the first paragraph is an overview.*

2. More clarification on the use of the term “safety” as a main result that participants describe is needed. Is it safety in a physical sense? Is it in an emotional sense? Safety in relation to what? To how they felt outside the unit? To their sense of dealing with their current condition? It is unclear in the opening paragraph. Indeed it is broken down and clarified later in the result section – but some short way to summarize the different aspects in the opening will be helpful.

*Again, thank you for pointing out the need to be clear and we have clarified what “safety” refers to.*

*“This contributed to a feeling of safety from risks of engaging in self harm or absconding.”*

Discussion
*The discussion can take a further step to integrate the findings back to SDT other contemporary works in MH. Specifying how they add (mostly by addressing the younger population it seems – and perhaps there are some nuances of the NZ culture and norms that are different from other countries or perhaps similar?
*The discussion is also the place where the authors may consider demonstrating how the findings can be interpreted in light of the SDT-oriented models they’ve been using, that may have supported the positive findings. This can be very interesting to readers to learn about.
Followed are some specific points and suggestions:
1.I would be more careful in stating that:
“Engaging young people in activities with a focus on relatedness, autonomy and competence may be more effective than talking or medication therapy”.

*As in response to Reviewer 1 we have removed this conclusion.*

However, perhaps one can definitely argue that in beginning stages where medication may be heavily sedative and unpleasant, or still has not reached fullest impact, the relational, SDT aspects are more important.

*This is a debatable issue and depends on the role one attributes to medication and to psycho-social interventions in treatment. It is beyond the focus of this paper.*

2.Citing how the current work adds to other attempts to veer psychiatric facilities to be more person centered may be also relevant (see for example, Ashcraft & Anthony, 2008, and there are more updated works in this area)

*Again, this is a very important and broad area. We have made a deliberate choice to focus the paper on Self-determination Theory and further discussion of other aspects of person-centred care are beyond the focus of this paper.*

[3.In](http://scanmail.trustwave.com/?c=7264&d=lJjH3lp2NKshGw06EGQNC8fZ13yPEeuEuxsgcbWUhw&u=http%3a%2f%2f3%2eIn) terms of the value of peer contact, and its relation to SDT in mental health, there is growing research in the area of adult peer support that might be relevant. See for example:
Moran, G. S., Russinova, Z., Yim, J.Y., Sprauge, C. (2014). Motivations of persons with psychiatric disabilities to work in mental health peer services: A qualitative study using Self-determination theory. Journal of Occupational Rehabilitation, 24(1):32-41
Or
Moran, G. S., Russinova, Z., Gidugu, V., Yim, J.Y., Sprauge, C. (2012). Benefits and mechanisms of peer providers with mental illnesses. Qualitative Health Research, 22(3), 304-319
And other works related to peers, compeers, peer specialists etc., by Cheryl Gagne, Mark Zalser, Mathew Chinman, Steve Gillard, Mike Slade.

*Thank you for this comment. We have added further detail about peer workers to the discussion.*

*“Peer support is well established in adult mental health care with some evidence of effectiveness in community care (Davidson, Bellamy, Guy, & Miller, 2012; Lloyd-Evans et al., 2014). Employment in peer mental health services also provides a career path for people with lived experience of severe mental illness (Moran, Russinova, Yim, & Sprague, 2014).”*

4. Clinical implication can perhaps also address how the models already used in your setting are support SDT, autonomy-supportive environments. Also enhancing training on person centered and goal centered interventions can further support SDT (see for example Hornik et al., 2018, and also Moran et al., Perceived assistance in pursuing personal goals and personal recovery among mental health residents across housing services. Psychiatry Research, 249, 94-101 – in it more relevant reference).

*Thank you for this suggestion too. It would be very valuable in a longer more general discussion paper of the area. However, we are favouring the approach of keeping the discussion focused tightly on the specific findings of this study.*

5.I would bring upfront to the opening paragraph of the discussion the part in the strengths that states:
his study adds to a small and limited pool of data. Most previous 604 studies are from longer stay units or residential treatment and few include young 605 people with psychotic illness. There are only two studies of units with average length 606 of stay less than 10 days (Grossoehme & Gerbetz, 2004; Moses, 2011). This is 607 important as the trend in inpatient care is towards shorter stays (Case, Olfson, 608 Marcus, & Siegel, 2007).”

*We agree that this is an important aspect of the paper but prefer to keep the structure as it is. We could be guided by the editor if that were to be changed*

All the best and good luck

6. PLOS authors have the option to publish the peer review history of their article ([what does this mean?](https://scanmail.trustwave.com/?c=7264&d=lJjH3lp2NKshGw06EGQNC8fZ13yPEeuEu0IlcuOSjA&u=https%3a%2f%2fjournals%2eplos%2eorg%2fplosone%2fs%2feditorial-and-peer-review-process%23loc-peer-review-history)). If published, this will include your full peer review and any attached files.


If you choose “no”, your identity will remain anonymous but your review may still be made public.


**Do you want your identity to be public for this peer review?** For information about this choice, including consent withdrawal, please see our [Privacy Policy](https://scanmail.trustwave.com/?c=7264&d=lJjH3lp2NKshGw06EGQNC8fZ13yPEeuEu0ItdbbAjg&u=https%3a%2f%2fwww%2eplos%2eorg%2fprivacy-policy).

Reviewer #1: No

Reviewer #2: Yes: Galia S. Moran


*To ensure confidentiality of participant confidentiality. We use quotations from the interviews to illustrate specific points as is common practice in qualitative research. All of the quotes cited in the texts were checked to ensure they contained no information which might identify participants. This ensured appropriate commitment to the expectation that participants’ identities would remain confidential.*
