# Supplementary material for: Self-determination theory in acute child and adolescent mental health inpatient care. A qualitative exploratory study
Source: PLoS One. 2020 Oct 16;15(10):e0239815. doi: 10.1371/journal.pone.0239815 (PMC7567378; doi:10.1371/journal.pone.0239815)
Supplement: Attachment — Submitted filename: PLOS ONE Response to reviewers 20 July 2020.docx [file pone.0239815.s002.docx]

Response to reviewers

Mean and median length of interviews.

We find this an odd request in a qualitative study like this but we have added a sentence stating, “The interviews lasted between 5 to 30 minutes with a mean length of 18.77 minutes and a median length of 14.45 minutes. These interviews generated 120 pages of transcript.”

The other issues raised by reviewers to address were those indentified by Reviewer 3 in the following paragraph:

Reviewer #3: This is an excellent piece of work that is described with great attention. I enjoyed reading the manuscript. It is informative and relatable. Authors generally seem to have followed COREQ guidelines while describing the study and its results. They have focused on relatedness, autonomy and competence from the perspectives of young people and their families. I would however like to recommend that authors expand the study aims as well as their rationale for the study design to be limited to SDT. It would be also useful to cover why an initial inductive followed by a deductive analytical approach was adopted by the authors. I gather that the intention was to understand SDT framework from the onset, hence possibly more direct and deeper information could have been collected if the interview guide was designed from a deductive or framework analysis perspective. In addition, given that these were inpatients actively seeking treatment during the course of the study and all the content is in relation to three SDT principles, authors may want to add some clinical implications of these findings and the non-SDT themes that may have been identified. Authors have addressed this briefly in the methods. However, I felt this argument could be strengthened further.

An initial inductive approach was used because we felt it was premature to focus the study questions solely on SDT which implied the assumption that these principles can be considered central to providing acute inpatient care. Therefore, we collected data broadly to allow the possibility that the We analyzed the data both openly and with an intentional focus on SDT. Most of what the young people spoke about did relate to the principles of SDT. We have made this clearer in the introduction to the results. With the following sentence:

“The only issues which were not able to be analysed within the SDT framework were some mixed feelings about medication and privacy and dissatisfaction with food.”

We agree with the reviewer that it would be appropriate to build on the findings of this exploratory study and focus more intensively on SDT in researching inpatient units.
