# Supplementary material for: Self-determination theory in acute child and adolescent mental health inpatient care. A qualitative exploratory study
Source: PLoS One. 2020 Oct 16;15(10):e0239815. doi: 10.1371/journal.pone.0239815 (PMC7567378; doi:10.1371/journal.pone.0239815)
Supplement: Attachment — Submitted filename: Hp PLOS ONE Resp to reviewers 3.9.20.docx [file pone.0239815.s003.docx]

Response to reviewers September 2 2020

There is one comment to address.

*Reviewer #3: I have no further comments except for a new observation around the additional information provided in the manuscript; relating to the length of the interviews. I see from the mean and median time (in my opinion could be given to just one decimal point), the interviews were rather short. Half of these were completed in below 15 minutes. I find that to be a major limitation. Perhaps the authors could comment about it in their limitations, providing reasons for such short interviews (clinical population, children, etc) and justify how confident they are about the richness of their qualitative content.*

On a previous review Reviewer #3 had requested the mean and median of the interview length. I thought this was unusual in a qualitative study but included them, to two decimal places, which as Reviewer #3 says, made no sense. Mean and median figures do not represent such a small number of interviews well, particularly given that there are two different sets of interviews. We have included a narrative description of the interviews which represents them better and included a statement about short interviews in the limitations section.

“Seven of the initial face to face interviews done in the unit were around 20 – 30 minutes with the rest ranging in length down to 5 minutes. Young people engaged in these to a variable degree. Some were difficult to engage and others talked freely. The follow up interviews were all shorter, six minutes or less and engagement was limited, with three of the young people saying little more than that they had nothing to add.”

“The interviews are short which is to be expected in interviews with adolescents in acute inpatient mental health care. The difference in length and engagement in the interviews done face to face in the unit and follow up by phone supports the approach to interviewing young people while in hospital despite the challenges involved in a safe consent process for this vulnerable population. The richness of qualitative data is inevitably limited in such short interviews, but this exploratory study opens important avenues for further study.”
